# Correction: Almutary et al. Development of 3D-Bioprinted Colitis-Mimicking Model to Assess Epithelial Barrier Function Using Albumin Nano-Encapsulated Anti-Inflammatory Drugs. *Biomimetics* 2023, *8*, 41

**DOI:** 10.3390/biomimetics10050265

**Published:** 2025-04-25

**Authors:** Abdulmajeed G. Almutary, Abdullah M. Alnuqaydan, Saleh A. Almatroodi, Hamid A. Bakshi, Dinesh Kumar Chellappan, Murtaza M. Tambuwala

**Affiliations:** 1 Department of Medical Biotechnology, College of Applied Medical Sciences, Qassim University, Buraydah 51452, Saudi Arabia; 2 Department of Medical Laboratories, College of Applied Medical Sciences, Qassim University, Buraydah 51452, Saudi Arabia; 3 The Hormel Institute, Medical Research Center, University of Minnesota, Austin, MN 55912, USA; 4 Department of Life Sciences, School of Pharmacy, International Medical University, Kuala Lumpur 57000, Malaysia; 5 Lincoln Medical School, University of Lincoln, Brayford Pool Campus, Lincoln LN6 7TS, UK

## 1. Error in Figure

In the original publication [1], there was a mistake in the published Figure 3, which had the following caption: “The manifestation in 3D-printed Caco-2 and HT-29 constructs. The 3D-printed Caco-2 and HT29 constructs were fixed in a 2% paraformaldehyde solution (10 mM calcium chloride, 50 mM sucrose in PBS) and paraffin embedded by using standard techniques. Using standard protocols, sections were stained with haematoxylin and eosin (H&E). (Scale bar: 20µm) Colitis manifestation was determined by viewing images under a microscope at 200×. (**A**,**B**) show untreated control 3D-printed CaCo-2 and HT-29 cell constructs, whereas (**C**,**D**) depict a DSS-induced colitis-like condition in 3D-printed Caco-2 and HT29 cellular constructs.” The figure mistakenly used a different source that was previously published. The corrected Figure 3 is provided below with the following caption: “Dextran sulphate sodium (DSS) on apical side of the cells disrupts epithelial cell barrier function, which, in turn, lower the resistance in the flow of electric current as measured using a TEER reader.”

## 2. Text Correction

There was an error in the original publication, particularly regarding the information provided in the histological images of Section 3.2. Also, Section 2.6, “*Histology*”, has been removed. The word “Measurements” was also added in Section 3.3, “*Barrier Function Measurements of 3D-Printed Caco-2 and HT-29 Constructs*”.

The following correction has been made to Paragraph 1 of Section 3.2:

### 3.2. Induction of Disease in a 3D-Printed Caco-2 and HT-29 Model

Caco-2 and HT-29 cells printed in a hydrogel using a 3D structure were treated with DSS 4% *w*/*v* for 24 h and showed a significant increase in epithelial disorganization/disfunction compared to the controls, which was assessed by the measurement of trans-epithelial electrical resistance (TEER), as depicted in Figure 3. This indicated that DSS induces a colitis-like condition in vitro [2].

## 3. References

Reference [2] has been newly added. Original ref. [20] was deleted. With this correction, the order of some references has been adjusted accordingly.

The authors state that the scientific conclusions are unaffected. This correction was approved by the Academic Editor. The original publication has also been updated.

## Figures and Tables

**Figure 3:**
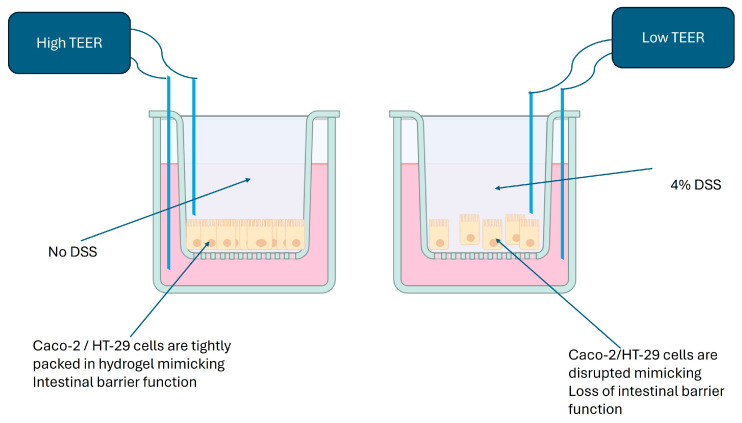
Dextran sulphate sodium (DSS) on apical side of the cells disrupts epithelial cell barrier function, which, in turn, lower the resistance in the flow of electric current as measured using a TEER reader.

## References

[1] Almutary A.G., Alnuqaydan A.M., Almatroodi S.A., Bakshi H.A., Chellappan D.K., Tambuwala M.M. (2023). Development of 3D-Bioprinted Colitis-Mimicking Model to Assess Epithelial Barrier Function Using Albumin Nano-Encapsulated Anti-Inflammatory Drugs. Biomimetics.

[2] Araki Y., Sugihara H., Hattori T. (2006). In vitro effects of dextran sulfate sodium on a Caco-2 cell line and plausible mechanisms for dextran sulfate sodium-induced colitis. Oncol. Rep..

